# Performance improvement and thermodynamic assessment of microchannel heat sink with different types of ribs and cones

**DOI:** 10.1038/s41598-022-14428-y

**Published:** 2022-06-24

**Authors:** Shizhong Zhang, Faraz Ahmad, Amjid Khan, Nisar Ali, Mohamed Badran

**Affiliations:** 1 Key Laboratory for Palygorskite Science and Applied Technology of Jiangsu Province, National and Local Joint Engineering Research Center for Mineral Salt Deep Utilization, Huaiyin Institute of Technology, Huai’an, 223003 China; 2 Department of Mechanical Engineering, Air University Islamabad, Aerospace and Aviation Campus, Kamra, 43570 Pakistan; 3 Mechanical and Aerospace Engineering Department, Oklahoma State University, Stillwater, USA; 4 Mechanical Engineering, Faculty of Engineering and Technology, Future University in Egypt, New Cairo, 11835 Egypt

**Keywords:** Engineering, Aerospace engineering, Mechanical engineering

## Abstract

The present study aims to investigate the performance of microchannel heat sink via numerical simulations, based on the first and second law of thermodynamics. The heat transfer and flow characteristics of rectangular microchannel heat sinks have been improved by adding six different types of surface enhancers. The cross-sections include rectangular, triangular, and hexagonal-shaped ribs and cones. The cones have been created from the same cross-sections of ribs by drafting them at an angle of 45° orthogonal to the base, which is expected to decrease the pressure drop, dramatically. The performance of ribs and cones has been evaluated using different parameters such as friction factor, wall shear stress, entropy generation rate, augmentation entropy generation number, thermal resistance, and transport efficiency of thermal energy. The results of the present study revealed that the novel effect of coning at an angle of 45° reduces frictional losses (Maximum pressure drop reduced is 85%), however; a compromise on thermal behavior has been shown (Maximum Nusselt number reduced is 25%). Similarly, the application of coning has caused a significant reduction in wall shear stress and friction factor which can lead to reducing the pumping power requirements. Moreover, triangular ribs have more ability to transfer thermal energy than rectangular and hexagonal ribs. Furthermore, it has been examined in the present study that the trend of total entropy generation rate for triangular ribs decreases up to Re = 400 and then increases onwards which means that thermal losses are more significant than frictional losses at lower Reynolds number. However, frictional losses dominate over thermal losses at higher Reynolds numbers, where vortex generation takes place, especially in triangular ribs.

## Introduction

The ever-growing technological advancements in integrated circuits have led to the generation of increasing heat flux as a result of heavy circuit accumulation in minimum size^1–5^. Consequently, it has resulted in the demand for efficient cooling techniques other than traditional ways. The rapid development in the field of micro-electromechanical systems has motivated researchers to develop new micro-cooling techniques. Numerous techniques have been developed previously including micro-heat pipe, micro-electro-hydrodynamic, and microchannel heat sink^6^. Among these techniques, the microchannel heat sink (MCHS) has proven to be the most efficient one. The study has been conducted for the first time by Tuckerman and Pease^7^ in 1981 showing heat transfer in silicon microchannel heat sink. The study was mainly focused on the ability of a microchannel heat sink to eliminate heat at the rate of 790 W/cm^2^. They depicted that greater area to volume surface provided by the heat sink has significantly increased the thermal efficiency. Microchannel heat sinks are the most advanced heat exchanging technologies incorporating the single-phase liquid flow. The applications of microchannel for single-phase liquid flow are the electronic devices cooling purposes, aerospace technology, and process equipment using laser technology^8^.

Ever since, the growing need for a microchannel heat sink, numerous experimental and numerical studies have been conducted to investigate the patterns of heat flow in a smooth rectangular microchannel. When it comes to improving the thermal performance of MCHS, there are several restrictions which put limitations such as pressure drop through the micro-channel as it adds to increase pumping power consumption and leakage risks. Furthermore, the small size of the channel makes the flow rigidly in the linear region leading to poor performance in comparison to irregular flow. With the continuous increase in loaded heat and attentive need for temperature measurement of electronic parts, the linear basic channel is hard to satisfy the need. Consequently, the focus of studies has been diverted towards passive methods and techniques that can be utilized to improve heat transfer performance in micro-channels. For example, Steinke and Kandlikar^9^ suggested several techniques that could come in handy for the enhancement of heat flow in micro-channels. One of the worth mentioning techniques is to incorporate the mixing features to enhance the flow of mixing, breakage of boundary surface to increase local heat transfer coefficient using fragmented construction.

In accordance with the above passive technique, many researchers have adopted such strategies to enhance the performance of micro-channel heat sink^10–13^. For example, Li et al.^14^ numerically investigated dimple and pin fin using microchannel heat sinks with water cooling, enhancement of heat transfer, reduction in resistance properties. Moreover, performance optimization has been noted in the new proposed design with the enlargement of diameter which in turn increases pin fin wake flow intensity and fierce flow in case of dimples. They revealed that flow separation occurs earlier in the case of dimples, so the wake flow is more intense in that case as com-pared to the pin fin case. Similarly, Rehman et al.^15^ investigated numerically the heat transfer and fluid flow behavior of microchannel heat sink by applying protrusions and dimples at different walls of the channel. They analyzed different designs, walls, and geometric configurations of these protrusions and dimples to optimize the performance of MCHS. Moreover, they varied pitch from 400 to 1200 µm and diameter of protrusions and dimples from 200 to 230 µm in a laminar flow regime having Re = 100–1000. Their results revealed that the addition of protrusion to all walls of MCHS (AWP) witnessed superior performance than all other cases in their study. Furthermore, they observed that AWP has achieved 115% improvement in the Nusselt number as compared to the smooth channel. Moreover, Li et al.^16^ proposed a hybrid heat sink consisting of metal foam (MF) and pin fins (PF) to solve the problem of high heating in electronic devices. Moreover, they also studied the effect of thermal contact resistance between MF and PF on the thermal performance of the metal foam pin fin (MFPFH) heat sink. They examined that the common contribution of pin fins and met-al foam can enhance both convection and conduction heat transfer behavior. Moreover, when they combined metal foam and pin fin (MFPFH), the performance ratio of MFPFH was 1.6 times higher than traditional PF, however; the Nusselt number of MFPFH was 36.3% and 266.6% higher than MF and PF heat sinks at a Reynolds number of 1000.

Many researchers^17–23^ investigated the effect of channel cross-section on the thermal and hydraulic behavior of a single phase MCHS. Compared to traditional de-signs like circular, triangular, and square, a rectangular channel has been proved a simple design for manufacturing simplicity with a reasonable thermal and hydraulic performance. In this manner, a lot of researchers have used a rectangular cross-section of the channel in their studies to investigate the performance of MCHS. For example, Li et al.^24^ investigated the thermodynamic behavior of MCHS with cavities of isosceles triangle shape. Moreover, they combined different fins with these cavities like forwarding drop-shaped fins, backward drop-shaped fins, streamlined fins, and rectangular fins. They evaluated the performance of MCHS based on thermodynamic analysis, field synergy principle, and thermal and hydraulic behavior. Their results indicated that the proposed models witnessed better heat transfer improvements be-cause of intensive secondary flows generation, boundary layer redevelopment, and better chaotic mixing relative to the conventional rectangular channel. Moreover, they revealed that these microstructures cause mainstream separation that leads to more friction factors and pressure drops. Therefore, they conducted overall performance which showed that the combination of the isosceles cavity with forwarding drop-shaped fin achieved the maximum thermal enhancement factor of 1.617 at a volume flow rate of 36 ml/min. Moreover, Hsieh et al.^25^ inspected microchannel by micro-particle image velocimetry flow visualization using incompressible liquid flow. They concluded that for Re < 200, pressure drop shows linear behavior but the pressure drops for Re > 200 shows nonlinear trend while fully developed flow regime visualization confirms the granular type of fluid flow in a microchannel.

Rehman et al.^26^ investigated the effect of novel sidewall ribs on the thermal and hydraulic performance of MCHS. In their study, the cross-sections of novel ribs included hydrofoil, trapezoid, ellipse, and rectangular ribs which were installed at side-walls of MCHS. The study was analyzed based on thermodynamic behavior including entropy generation and transport efficiency of thermal energy in a laminar flow regime of Reynolds number from 100 to 1000. They concluded that among all four cross-sections, hydrofoil ribs performed better having the lowest augmentation entropy generation number. Chai et al.^27^ studied the effect of geometric parameters of triangular reentrant cavities on water flow and heat transfer features in microchannel heat sinks using numerical simulations. Their research concluded that convective fluid mixing can be greatly enhanced by wakes in triangular reentrant cavities that result in disordered advection. The heat transfer enhancement in the constant cross-section segment can occur by the repeated developing flow and thermal and hydraulic boundary layers interruption. Moreover, they observed that the intensity of the vortex can be enhanced by appropriate improvement of Reynolds number in compensation of pressure drop. Moreover, Xia et al.^28^ numerically investigated the effect of structural parameters of fan-shaped reentrant cavities on pressure drop and thermal resistance, which includes the constant cross-section region and arcuate region lengths and widths. The fan-shaped reentrant cavities enhanced the heat transfer by improving the boundary layer, increment in surface area, and the wake/jet effect. The intensity of each effect depends upon the flow rate and the number of fan-shaped reentrant cavities. Ghani et al.^29^ proposed a new design of microchannel with rectangular ribs and sinusoidal cavities and numerically investigated the fluid flow and thermal characteristics with various Reynolds numbers from 100 to 800. They observed that the proposed design of the microchannel performs well in terms of friction factor, Nusselt number, and performance factor. Moreover, the performance factor achieved in this study was 1.85 as compared to the smooth channel.

Ahmad et al.^30^ investigate the behavior of MCHS by introducing rectangular, triangular, and hexagonal ribs at all walls of the channel. They revealed that these ribs had a better heat transfer performance with a penalty of high-pressure drops. In the same study, they proposed that the high-pressure drop of these ribs can be decreased by applying a novel technique of coning. They drafted the cross-sections by 450 angles to make them cone-like ribs. They examined that there was a huge pressure drop with a little compensation of heat transfer characteristics. Similarly, Wang et al.^31^ fabricated microscale ribs and grooves on the heated wall of MCHS and carried out an experimental and numerical study to analyze the effect of ribs and grooves on cooling effectiveness. They observed that the Nusselt number was improved about 1.11 to 1.55 times for the proposed design when compared to a smooth microchannel. However, at the penalty of high-pressure drop. Moreover, they concluded that for a relative rib height of 0.85, the friction factor of rib grooved MCHS was 4.09 times higher than the smooth channel. Similarly, six different configurations of ribbed microchannel were numerically investigated for the Reynolds number in the range of 100–500 by Khan et al.^32^. They concluded that triangular ribs show low thermal resistance among the six different configurations for the above range of Reynolds number, however; rectangular ribs induce high-pressure drop. Furthermore, Zhai et al.^33^ investigated fluid flow distribution, fluid temperature, and thermal resistances in microchannel and presented an empirical model. In addition to that, they observed that fluid flow with uniform distribution results in an equally distributed thermal field at the bottom surface of the microchannel.

Many studies have been focused on thermodynamic analysis based on entropy generation^34–38^ and exergy analysis^39–42^. However, to the best of the author’s knowledge, no work has been done to investigate the thermodynamic behavior of such types and the orientation of ribs and to compare their performance with their corresponding conical geometries. In this study, three different types of rib cross-sections have been used to improve the thermodynamic performance of MCHS by installing these ribs on the centerline of each wall of the channel. These rib cross-sections include triangular, rectangular, and hexagonal geometry. Moreover, these three cross-sections have been drafted at an angle of 45° to make them a cone-like structure to reduce their drag on flow which is expected to reduce the pumping power requirements. The performance of all the aforementioned cases has been evaluated using different parameters based on the first and second laws of thermodynamics. The performance evaluation parameters include friction factor, wall shear stress, entropy generation rate, augmentation entropy generation number, thermal resistance, and transport efficiency of thermal energy.

## Physical model description

**Figure 1:**
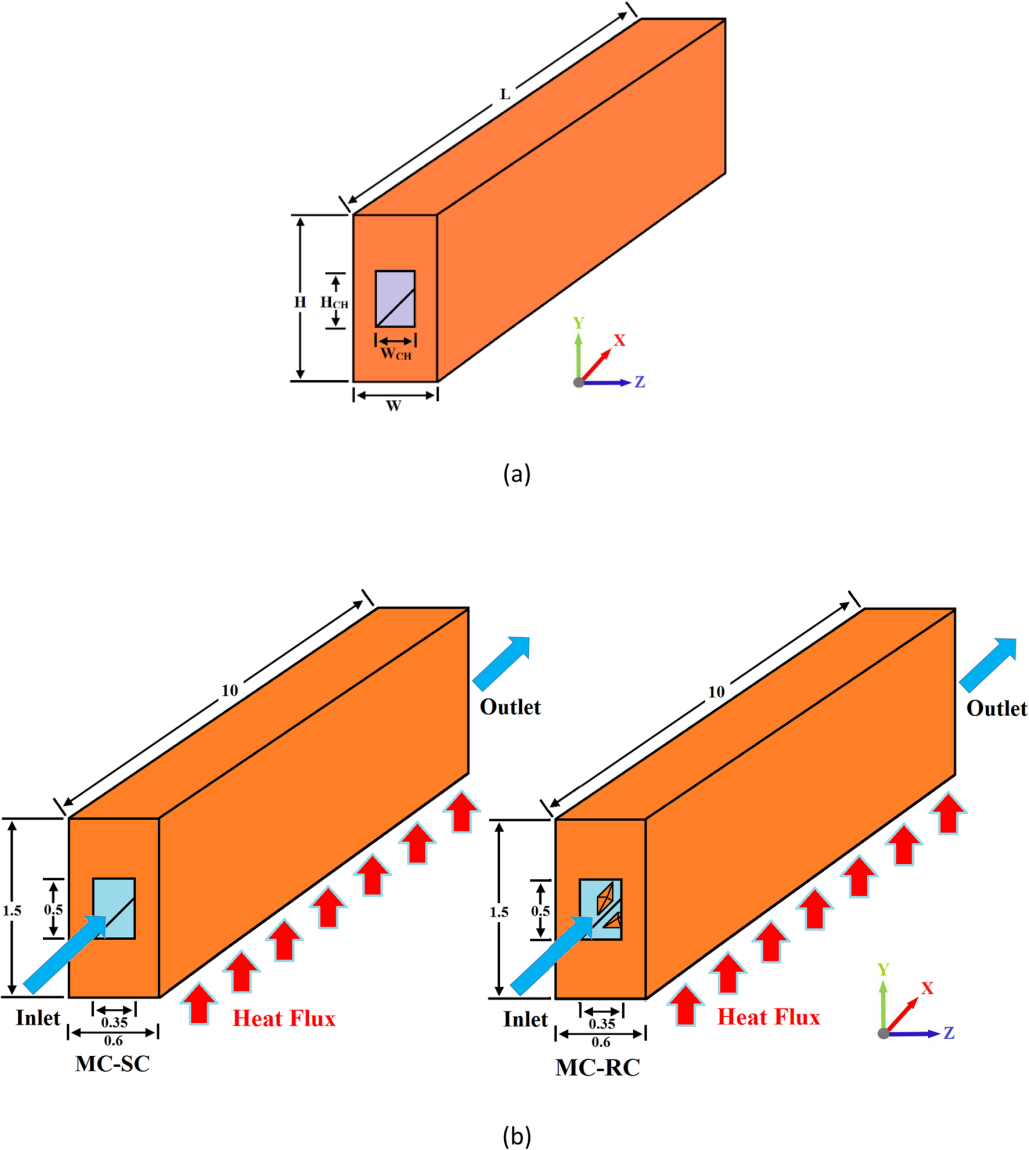
(**a**) Computational domain of MCHS (**b**) 3D geometry of MCHS with smooth channel (MC-SC) and rectangular cones (MC-RC).

**Figure 2:**
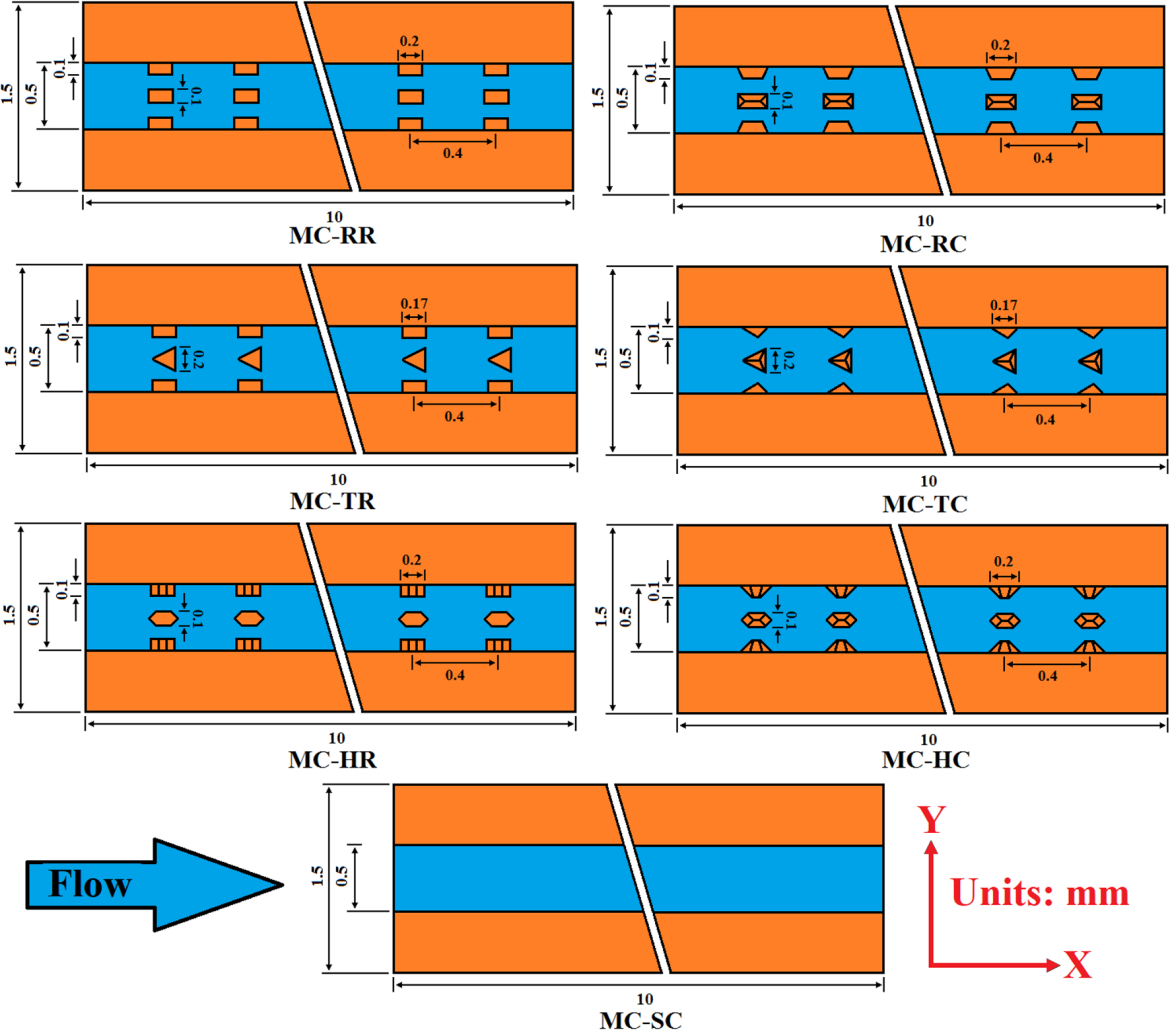
Comparison of sectioned geometry of all types of ribs and cones in the x–y plane.

In the present study, a microchannel heat sink has been modeled to investigate its thermodynamic behavior, numerically. Moreover, MCHS with smooth channel (MC-SC) has been compared with six different types of ribs and cones to enhance its hydrothermal properties. The ribs and cones involved in the present study include rectangular ribs (MC-RR), triangular ribs (MC-TR), hexagonal ribs (MC-HR), rectangular cones (MC-RC), triangular cones (MC-TC), and hexagonal cones (MC-HC). Figure 1a shows the computational domain of MCHS and Fig. 1b shows the 3D geometry of MC-SC and MC-RC which represents the dimensions of the solid domain (copper) and fluid domain (water). Similarly, MC-RC shows the orientation of rectangular cones in the direction of flow that predicts the orientation of other ribs and cones. The detailed geometric parameters are listed in Table 1. Moreover, for the sake of simplicity and further details, a 2D cross-section of all cases has been shown in Fig. 2 which also represents the dimensions and spacing of ribs/cones. Furthermore, the thermo-physical properties of all materials used in the present study are given in Table 2.

**Table 1:** List of geometric parameters used in computational domain.

Parameters	Symbols	Values (µm)
Length of MCHS	L	10,000
Height of MCHS	H	1500
Width of MCHS	W	600
Hydraulic diameter of channel	D_h_	412
Height of rectangular microchannel	Hch	500
Width of rectangular microchannel	Wch	350
Height of rib	Hr	100

**Table 2:** Thermo-physical properties of materials.

Materials	Density $(\rho ) \left(\mathrm{kg}/{\mathrm{m}}^{3}\right)$	Specific heat $\left({c}_{p}\right)$ $(\mathrm{J}/\mathrm{kg}/\mathrm{K})$	Thermal conductivity $\left(\mathrm{k}\right) \left(\mathrm{W}/\mathrm{m}/\mathrm{K}\right)$	Dynamic viscosity $\left(\mu \right)\left(\mathrm{kg}/\mathrm{m}/\mathrm{sec}\right)$
Copper	8978	381	387.6	–
Water	998.2	4182	0.6	0.001003

## Mathematical model description

### Assumptions

The following assumptions were made before solving the mathematical model in the present study:

1. The flow was considered as continuum to validate for no slip condition.
2. A laminar flow with Re = 100–1000 with steady state and incompressible flow.
3. The thermo-physical properties were considered as independent of temperature for both solid and liquid domain.
4. The effects of gravity and radiation has been ignored.

### Governing equations

After considering the above assumptions, the following governing equations are used in the mathematical modelling:

*Continuity Equation*:

1 $$\frac{\partial u}{\partial x}+\frac{\partial v}{\partial y}+\frac{\partial w}{\partial z}=0$$

where, *u*, *v*, and *w* show components of velocity in *x*, *y* and *z* direction.

*Momentum Equations*:

*X-Momentum*

2 $$u\frac{\partial u}{\partial x}+v\frac{\partial u}{\partial y}+w\frac{\partial u}{\partial z}=-\frac{1}{{\rho }_{f}}\frac{\partial p}{\partial x}+\frac{{\mu }_{f}}{{\rho }_{f}}\left(\frac{{\partial }^{2}u}{\partial {x}^{2}}+\frac{{\partial }^{2}u}{\partial {y}^{2}}+\frac{{\partial }^{2}u}{\partial {z}^{2}}\right)$$

*Y-Momentum*

3 $$u\frac{\partial v}{\partial x}+v\frac{\partial v}{\partial y}+w\frac{\partial v}{\partial z}=-\frac{1}{{\rho }_{f}}\frac{\partial p}{\partial y}+\frac{{\mu }_{f}}{{\rho }_{f}}\left(\frac{{\partial }^{2}v}{\partial {x}^{2}}+\frac{{\partial }^{2}v}{\partial {y}^{2}}+\frac{{\partial }^{2}v}{\partial {z}^{2}}\right)$$

*Z-Momentum*

4 $$u\frac{\partial w}{\partial x}+v\frac{\partial w}{\partial y}+w\frac{\partial w}{\partial z}=-\frac{1}{{\rho }_{f}}\frac{\partial p}{\partial z}+\frac{{\mu }_{f}}{{\rho }_{f}}\left(\frac{{\partial }^{2}w}{\partial {x}^{2}}+\frac{{\partial }^{2}w}{\partial {y}^{2}}+\frac{{\partial }^{2}w}{\partial {z}^{2}}\right)$$

where ${\mu }_{f}$, *p*, and ${\rho }_{f}$ represents dynamic viscosity, pressure, and density of fluid domain, respectively.

*Energy Equations:*

For Fluid domain,

5 $$u\frac{\partial {T}_{f}}{\partial x}+v\frac{\partial {T}_{f}}{\partial y}+w\frac{\partial {T}_{f}}{\partial z}=\frac{{k}_{f}}{{{\rho }_{f}C}_{Pf}}\left(\frac{{\partial }^{2}{T}_{f}}{\partial {x}^{2}}+\frac{{\partial }^{2}{T}_{f}}{\partial {y}^{2}}+\frac{{\partial }^{2}{T}_{f}}{\partial {z}^{2}}\right)$$

where ${C}_{Pf}$, ${T}_{f}, \mathrm{and} {k}_{f},$ represent specific heat, fluid temperature, and thermal conductivity of fluid domain.

For Solid domain,

6 $${k}_{s}\left(\frac{{\partial }^{2}{T}_{s}}{\partial {x}^{2}}+\frac{{\partial }^{2}{T}_{s}}{\partial {y}^{2}}+\frac{{\partial }^{2}{T}_{s}}{\partial {z}^{2}}\right)=0$$

where ${T}_{s}$ and ${k}_{s}$ represents temperature and thermal conductivity of solid domain.

### Boundary conditions

To simplify the above governing equations, following boundary conditions are considered:

At outlet boundary of the channel, ambient pressure is applied. Where;

7 $$x=10\, \mathrm{mm}; p={p}_{out}=1 \,\text{atm}$$

At inlet boundary of the channel, a uniform velocity and temperature conditions are given. Where;

8 $$x=0; u={u}_{in}$$

9 $${T}_{f}={T}_{in}=293.15\,\text{K}$$

At the bottom wall, a constant heat flux is given. Where;

10 $$y=0 : -{k}_{s}\frac{\partial {T}_{s}}{\partial y}={q}_{w}=100\, \mathrm{W}/{\mathrm{cm}}^{2}$$

No slip condition was given at solid–liquid interface for both thermal and hydrodynamic behavior. Where;

11 $$-{k}_{s}\frac{\partial {T}_{s}}{\partial n}=-{k}_{f}\frac{\partial {T}_{f}}{\partial n}$$

12 $$u=v=w=0$$

where *n* represents normal to the channel wall.

At top surface of the channel, adiabatic boundary condition is given.

13 $$\frac{\partial {T}_{s}}{\partial n}= \frac{\partial {T}_{f}}{\partial z}=0$$

Symmetry boundary conditions were applied at the rest sides.

### Definition of evaluating parameters

Reynolds number (*Re*) has been calculated from mean velocity ${u}_{m}$ as:

14 $$Re=\frac{{\rho }_{f}{u}_{m}{D}_{h}}{{\mu }_{f}}$$

where ${D}_{h}$ represents hydraulic diameter. For non-circular ducts, hydraulic diameter can be defined as the ratio of area (A) and wetted perimeter (P) as:

15 $${D}_{h}=\frac{4A}{P}$$

For a rectangular cross-section, it can be given in the form of channel width (W_ch_) and height (H_ch_) simplified as:

16 $${D}_{h}=\frac{2{H}_{ch}{W}_{ch}}{{H}_{ch}+{W}_{ch}}$$

The Average Euler number (*Eu*) is given as:

17 $$Eu=\frac{{P}_{in}-{P}_{out}}{{{{\rho }_{f}u}_{m}}^{2}}$$

The average friction factor $f$ can be calculated using pressure drop ($\Delta p$) along the channel length (L):

18 $${f}_{app}=\frac{2{D}_{h}\Delta p}{L{\rho }_{f}{u}_{{m}^{2}}}$$

By definition, wall shear stress is directly proportional to gradient of velocity near the channel wall in the direction normal to it i.e. y and z direction in the present study.

19 $${\tau }_{w}=\mu {\left(\frac{d{u}_{j}}{d{x}_{i}}\right)}_{d{x}_{i}=0}$$

where ${\tau }_{w}$ represents wall shear stress, $\mu$ is the dynamic viscosity, *u*_*j*_ is the velocity normal to *x*_*i*_ direction. As the Newtonian fluid flows under steady conditions in rigid wall channel, the flow is fully developed i.e. the velocity is maximum at center and zero at walls^43^. Such type of flow is usually called as poiseuille flow and the wall shear stress for this flow can be given as:

20 $${\tau }_{w}=\frac{4\mu {u}_{max}}{{D}_{h}}$$

The average Nusselt number (Nu) can be calculated from the below heat transfer coefficient (h) as^31^:

21 $$h=\frac{{q}_{w}{A}_{b}}{2\left({W}_{ch}+{H}_{ch}\right){L}_{ch}\Delta T}$$

22 $$Nu=\frac{h{D}_{h}}{{k}_{f}}$$

where A_b_ is the area of bottom wall and $\Delta T$ is the average temperature difference between channel wall and fluid.

23 $$\Delta T={T}_{w}-{T}_{f}$$

24 $${T}_{w}=\frac{\int{T}_{w-x,y}dydx}{\int dydx}$$

25 $${T}_{f}=\frac{\int{T}_{f-i,x}{\rho }_{f-i,x }\left|\overrightarrow{v}.\overrightarrow{dA}\right|dx}{\int{\rho }_{f-i,x }\left|\overrightarrow{v}.\overrightarrow{dA}\right|dx}$$

The total thermal resistance is sum of convective resistance (*R*_*conv*_), conductive (*R*_*cond*_), and capacitive (*R*_*cap*_) resistance as:

26 $${R}_{tot}={R}_{cond}+{R}_{conv}+{R}_{cap}$$

27 $${R}_{tot}=\frac{{T}_{b}-{T}_{w}}{{q}_{w}{A}_{b}}+\frac{{T}_{w}-{T}_{f}}{{q}_{w}{A}_{b}}+\frac{{T}_{f}-{T}_{in}}{{q}_{w}{A}_{b}}=\frac{{T}_{b}-{T}_{in}}{{q}_{w}{A}_{b}}$$

where *T*_*b*_ represents temperature of the base wall that can be given as:

28 $${T}_{b}=\frac{\int {T}_{b-x,y}dydx}{\int dydx}$$

The irreversibility is occurring because of many main reasons in which the most important two are; (1) heat transfer and (2) friction or pressure drop. The irreversibility of heat transfer has been represented by thermal entropy generation rate (${\dot{S}}_{gen,\Delta T}$) and that of friction has been represented by frictional entropy generation rate (${\dot{S}}_{gen,\Delta P}$) in the present study. The total entropy generation rate can be calculated by summing both thermal and frictional entropy generation rates as given by Zhai et al.^44^:

29 $${\dot{S}}_{gen,Tot}={\dot{S}}_{gen,\Delta T}+{\dot{S}}_{gen,\Delta P}$$

30 $${\dot{S}}_{gen,\Delta T}=\frac{{k}_{f}}{{T}^{2}}\left[{\left(\frac{\partial T}{\partial x}\right)}^{2}+{\left(\frac{\partial T}{\partial y}\right)}^{2}+{\left(\frac{\partial T}{\partial z}\right)}^{2}\right]$$

31 $${\dot{S}}_{gen,\Delta P}=\frac{{\mu }_{f}}{T}\left\{2\left[{\left(\frac{\partial u}{\partial x}\right)}^{2}+{\left(\frac{\partial v}{\partial y}\right)}^{2}+{\left(\frac{\partial w}{\partial z}\right)}^{2}\right]+{\left(\frac{\partial u}{\partial y}+\frac{\partial v}{\partial x}\right)}^{2}+{\left(\frac{\partial u}{\partial z}+\frac{\partial w}{\partial x}\right)}^{2}+{\left(\frac{\partial v}{\partial z}+\frac{\partial w}{\partial y}\right)}^{2}\right\}$$

To compare the irreversibility of enhanced channels with that of smooth channel, Bejan^45^ has defined augmentation entropy generation number (*N*_*s*_), which is the ratio of entropy generation of any enhanced channel (${\dot{S}}_{gen}$) to that of smooth channel (${\dot{S}}_{gen,0}$).

32 $${N}_{s}=\frac{{\dot{S}}_{gen}}{{\dot{S}}_{gen,0}}$$

It is well known that thermal energy has relatively low grade comparative to electrical, chemical, and mechanical energies. Therefore, much of its part get wasted in transfer process. It is therefore seems essential to analyze its effective utilization based on second law of thermodynamics which is also called as exergy analysis. The parameter used for exergy analysis in the present study is transport efficiency which was first presented by Liu et al.^46^.

33 $${\upeta }_{t}=1-\frac{{\dot{Q}}_{d}}{\dot{Q}}$$

where Q_d_ represents irreversible heat loss and is given as:

34 $${\dot{Q}}_{d}={\iiint }_{\Omega }\frac{{k}_{f}{\left(\nabla {T}_{f}\right)}^{2}}{{T}_{f}}dV$$

## Computational model description

### Numerical code and convergence criteria

A three dimensional conjugate heat transfer has been solved using the available CFD (computational fluid dynamics) code of ANSYS (FLUENT) 15.0. The analysis used a finite volume based model to solve the governing equations of continuity, momentum, and energy. The convective part of governing equations has been discretized by QUICK and diffusive part by second-order upwind interpolation which results in rapid convergence of the model. Moreover, SIMPLE algorithm has been used for coupling of pressure and velocity.

The solution is considered to be converged when the residuals of continuity, momentum and energy equations become less than 10^–6^ as given below:

35 $$\sum_{i=0}^{{N}_{x}}\sum_{j=0}^{{N}_{y}}\sum_{k=0}^{{N}_{z}}\left|V\left(i,j,k\right)-{V}_{0}(i,j,k)\right|\le {10}^{-6}$$

36 $$\sum_{i=0}^{{N}_{x}}\sum_{j=0}^{{N}_{y}}\sum_{k=0}^{{N}_{z}}\left|P\left(i,j,k\right)-{P}_{0}(i,j,k)\right|\le {10}^{-6}$$

37 $$\sum_{i=0}^{{N}_{x}}\sum_{j=0}^{{N}_{y}}\sum_{k=0}^{{N}_{z}}\left|T\left(i,j,k\right)-{T}_{0}(i,j,k)\right|\le {10}^{-6}$$

where *N*_*x*_*, N*_*y*_*, and N*_*z*_ are number of grids along *x, y and z* directions.

### Mesh independence analysis

**Figure 3:**
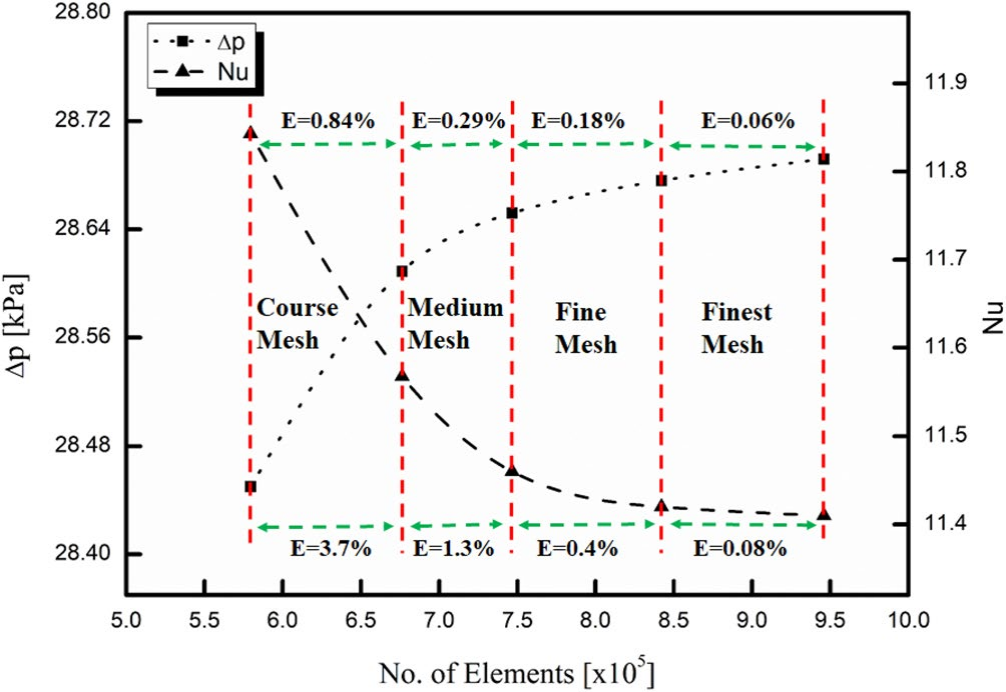
Mesh independence analysis.

The present study was totally based on numerical simulations using Fluent 15.0. Therefore, it was considered to check the dependency of mesh size on simulation results. For this sake, four different ranges of mesh size using a hexahedral mesh as the base unit were designed and analyzed. The ranges of mesh size were comprised of course mesh (5.8 × 10^5^–6.8 × 10^5^), medium mesh (6.8 × 10^5^–7.5 × 10^5^), fine mesh (7.5 × 10^5^–8.5 × 10^5^), and finest mesh (8.5 × 10^5^–9.5 × 10^5^). The relative error between finest mesh and any other mesh can be calculated using below formula.

38 $$E=\left|\frac{{M}_{2}-{M}_{1}}{{M}_{1}}\right|\times 100\%$$

where M_1_ represents results for finest mesh and M_2_ represents results for any mesh other than finest. The mesh independence was checked for both thermal (Nusselt number) and hydraulic (pressure drop) behavior of MCHS as shown in Fig. 3. It was observed that results get closer to finest mesh when the mesh size was increased gradually. Moreover, it can be seen that relative error became less than 1% for both Nusselt number and pressure drop in the range of fine mesh. Therefore, this range was selected for further analysis to achieve accuracy and save computational time.

### Validation of numerical code

**Figure 4:**
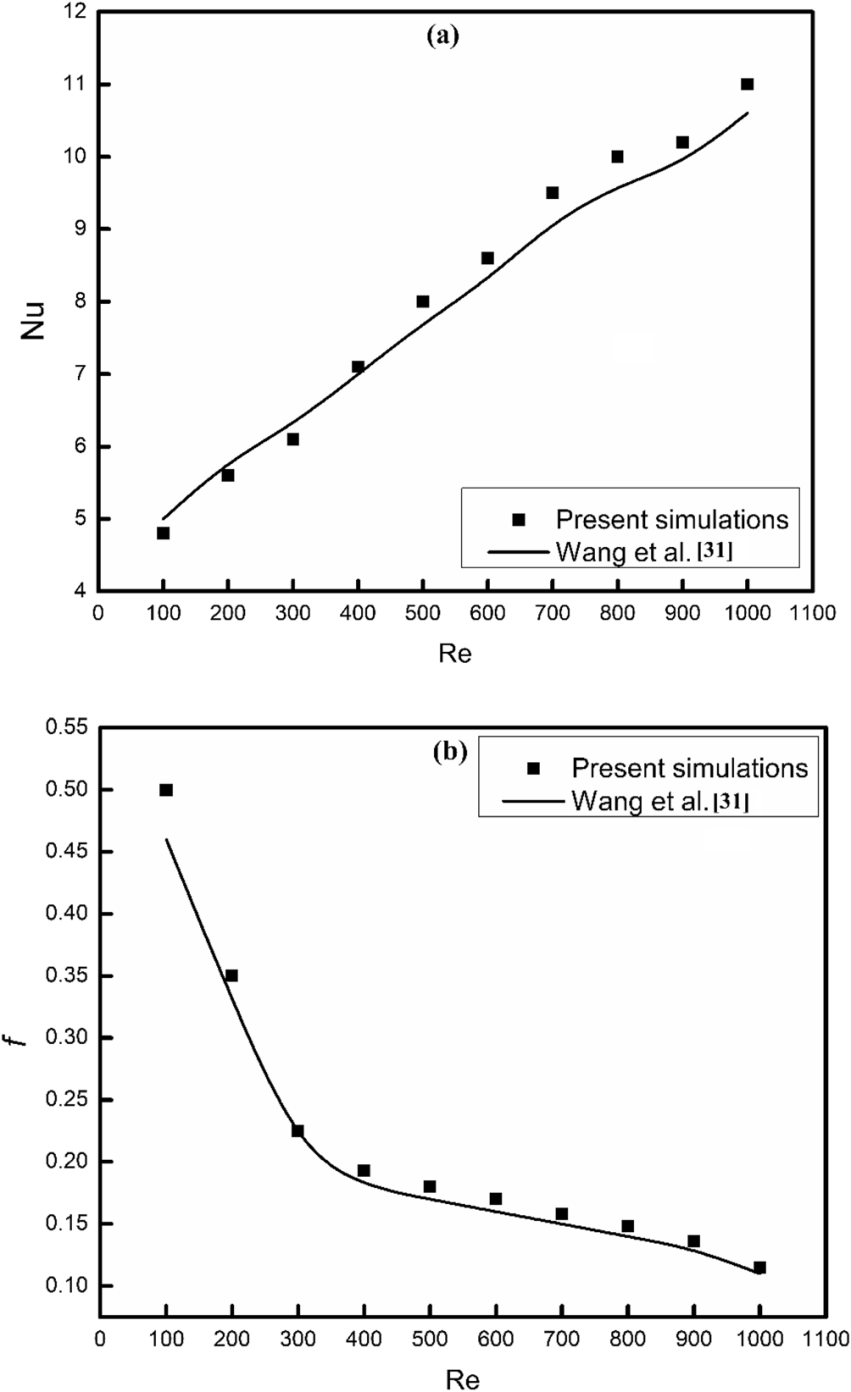
Validation of the present numerical simulations.

To check the further accuracy of the present numerical simulations, results of the present study have been validated with the available experimental results of Wang et al.^31^. It was noticed that the relative difference between present numerical simulations and Wang et al.^31^ was much lesser than 10% for both friction factor and Nusselt number as shown in Fig. 4. Therefore, the present numerical model can be further used for the investigation of flow and thermal behavior of MCHS.

## Results and discussion

In the present study, three dimensional numerical simulations have been done to investigate the effect of ribs and cones installed at the centerline of all walls of MCHS. The study was conducted by applying a constant heat flux of 100 W/cm^2^ under a laminar flow condition of having Reynolds number from 100 to 1000. The results of the study were analyzed by considering hydrodynamic behavior, thermal behavior, entropy generation analysis, and exergy analysis of MCHS. The reliability of numerical code has been verified by mesh independence analysis and validation with already existing experimental results in the literature.

### Hydrodynamic analysis

Hydrodynamic behavior of all cases in the present study has been investigated by comparing their friction factor and wall shear stress.

**Figure 5:**
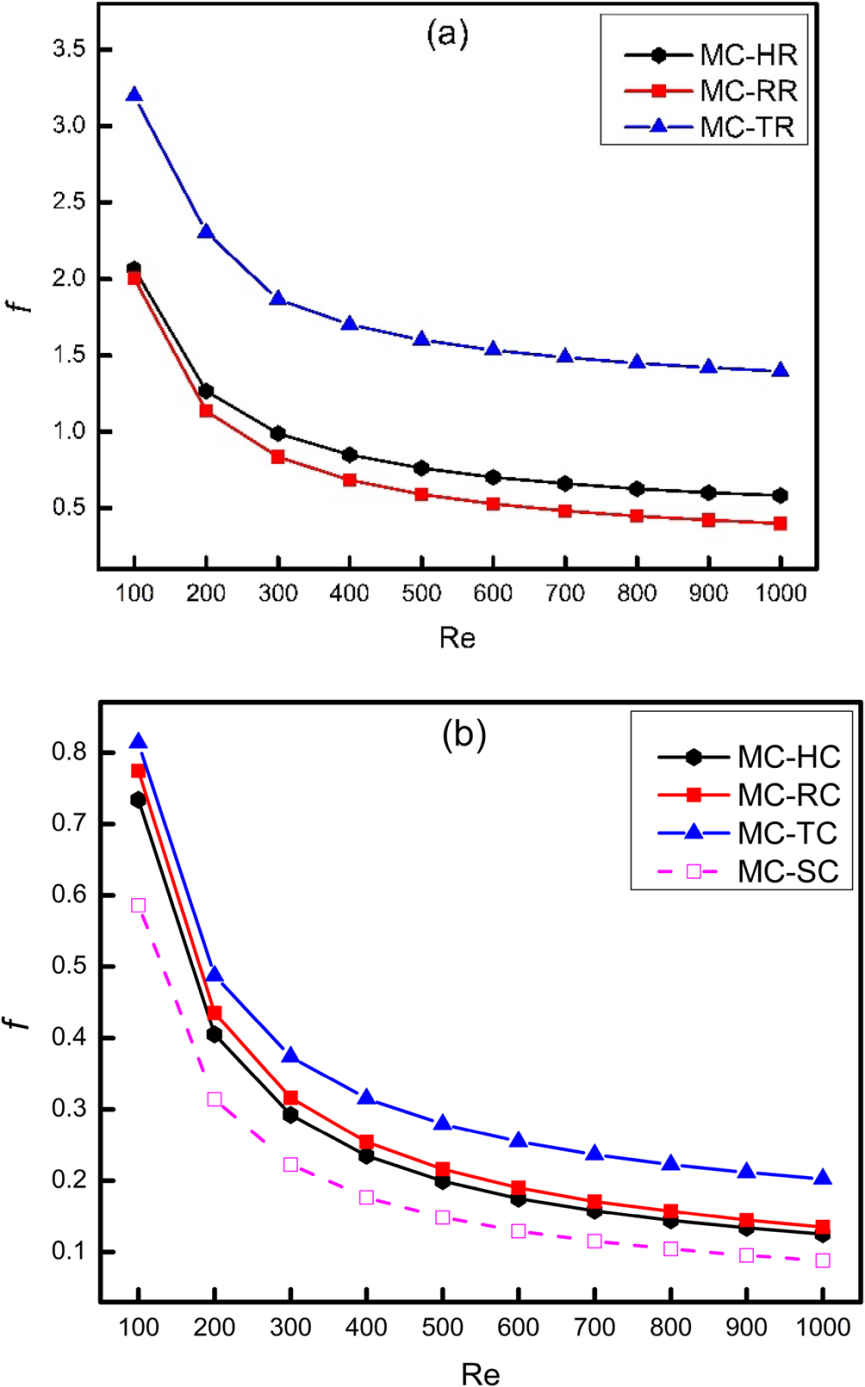
Variation of friction factor with Reynolds number.

The variation of friction factor with Reynolds number has been shown in Fig. 5 where part (a) compares friction factor of rectangular, triangular and hexagonal ribs and part (b) compares friction factor of corresponding cones. It is obvious that friction factor decreases with increase in Reynolds number because the friction factor is arising from viscosity of fluid between layers and this resistance in different layers of fluid decreases with increasing velocity. In simple words, the increase in Reynolds number diminishes the effect of viscous sub layer dissipation. It has been clarified that triangular ribs have maximum friction factor because of having more frontal area. The more frontal area of triangular ribs offers more resistance to flow as compared to rectangular and hexagonal ribs, which leads to more pressure drop. Moreover, the friction factor of hexagonal ribs is more than rectangular ribs because of having more number of corners available in hexagonal rib than rectangular rib. For the sake of reduction in this huge pressure drop, the edges of all these ribs have been drafted at an angle of 45^0^ to make them conical like shapes which can serve as a streamlining action. The action of streamlining causes a reduction in flow blocking which results in a lower pressure drop as compared to ribs structure. The aforementioned effect is very clear and has been shown in Fig. 5b.

It is a common concept that when a fluid flows inside a stationary channel, the velocity of fluid is different at different points which is called as velocity gradient. Also, it is clear that velocity is maximum at the center and minimum at the channel walls. This gradient of velocity occurs because of frictional forces which occurs either between the fluid layers or between the solid wall and the adjacent fluid layer to it. The former effect arises because of viscosity and the later arises because of roughness of the wall. Because of these friction forces, there is a tangential force acting per unit area which is exerted by the walls of channel on fluid layers and that is called as wall shear stress.

**Figure 6:**
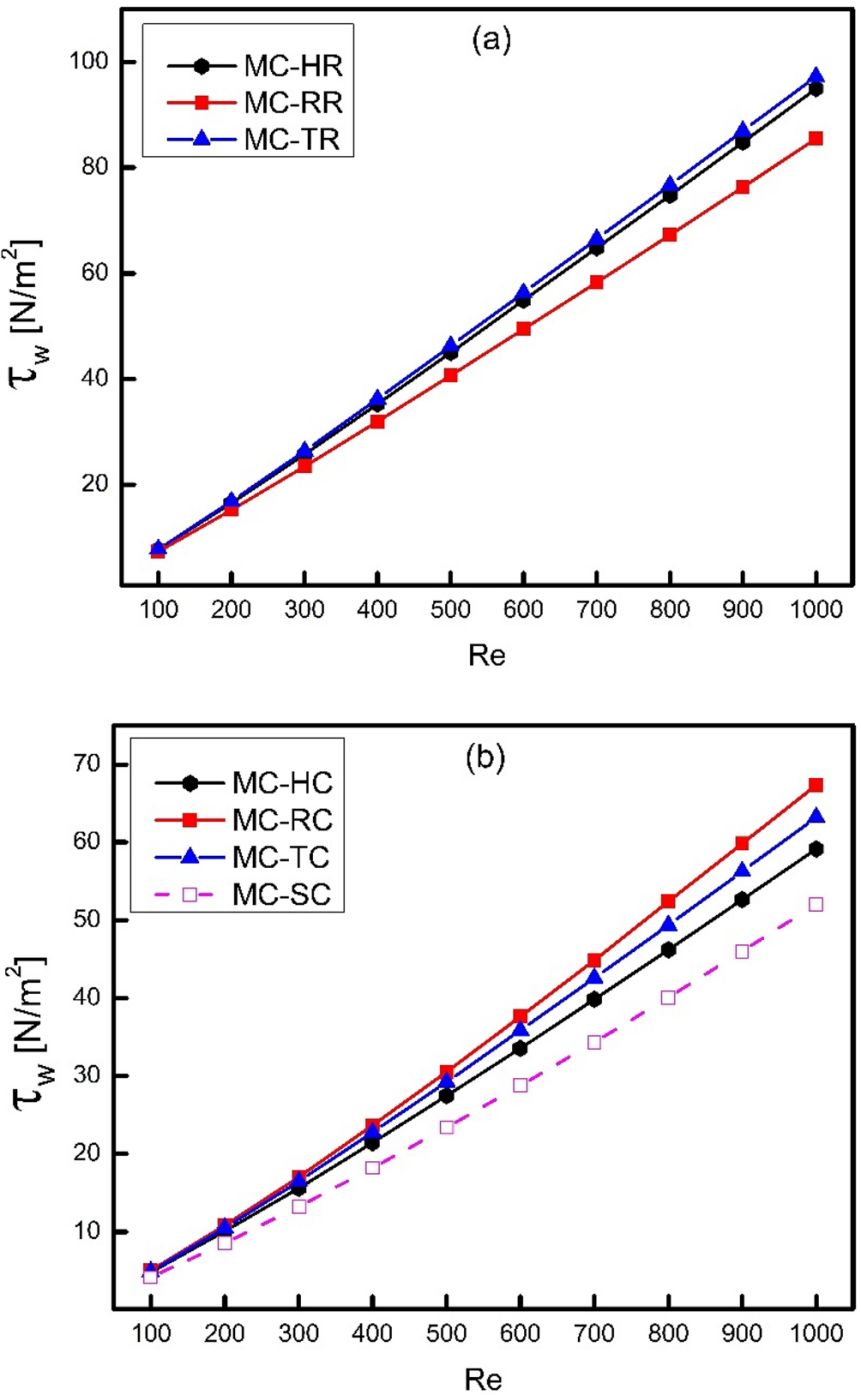
Variation of wall shear stress with Reynolds number.

Figure 6 shows the variation of wall shear stress with Reynolds number where part (a) of the figure gives a comparison of wall shear stress for micro channels having ribs on their walls. It can be seen that wall shear stress increases with increase in Reynolds number and it has been noticed that triangular ribs have maximum wall shear stress. However, rectangular ribs have minimum wall shear stress because of its lower frontal area which offers lesser friction than triangular and hexagonal ribs. Moreover, Fig. 6b shows the comparison of wall shear stress for drafted ribs (cones). The difference is very obvious in wall shear stress of ribs and cones where cones have showed a clear reduction in wall shear stress as compared to ribs. The reduction in wall shear stress for cones is because of streamlining action achieved by cones which reduces friction drastically and thus shear stress. In all the three types of cones, the rectangular cones have maximum shear stress while hexagonal ribs have minimum shear stress. Moreover, the shear stress of smooth channel is the lowest in all cases which is obvious because of smooth walls offering lesser friction as compared to rough channels.

### Thermal analysis

**Figure 7:**
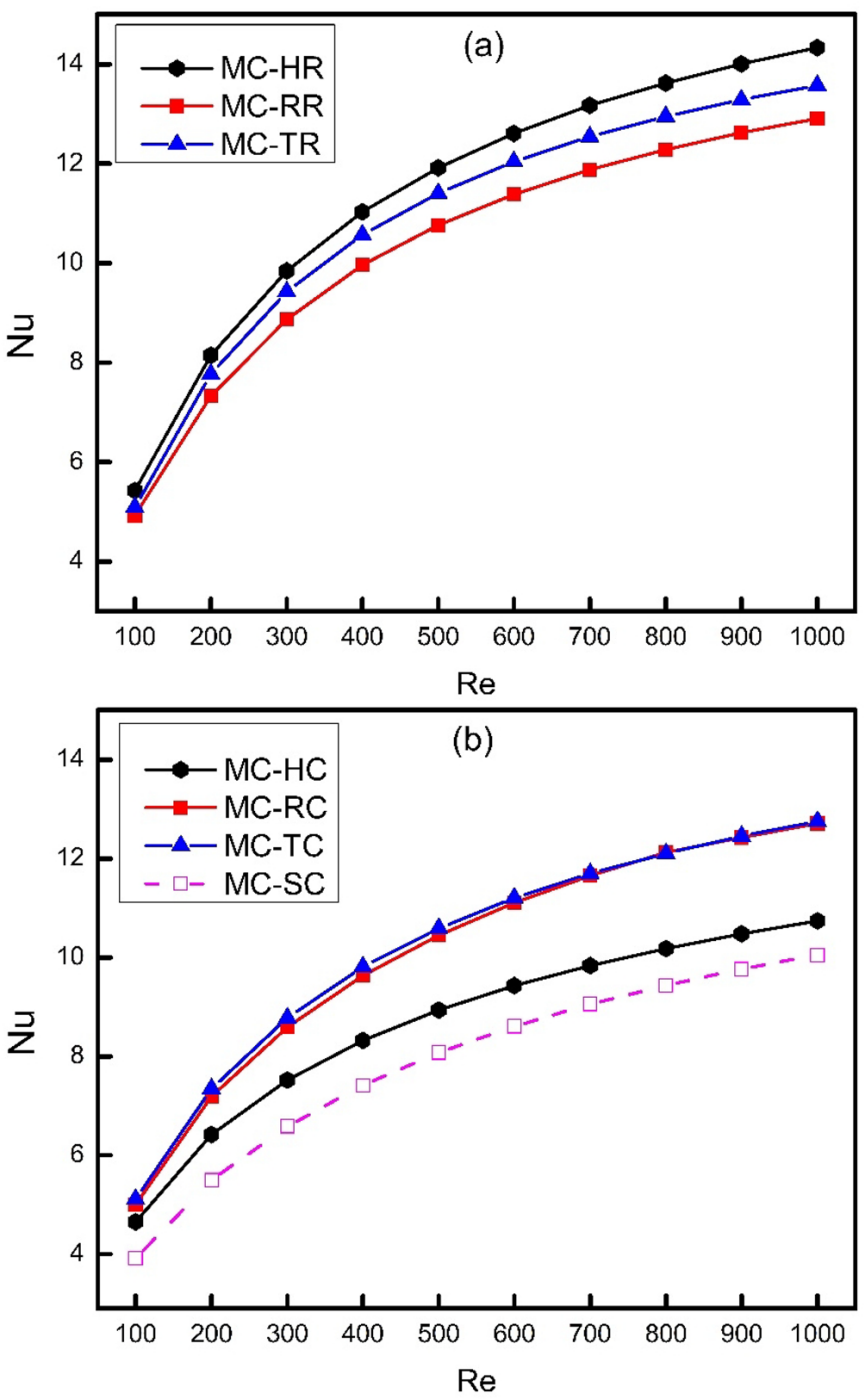
Variation of Nusselt number with Reynolds number.

Figure 7 demonstrates the effect of increasing Reynolds number on heat transfer behavior of different MCHS with and without surface enhancers (ribs and cones) in the form of Nusselt number (Nu). It is obvious from the figure that Nu increases as the Reynolds number increases because the convection heat transfer has a direct relation with fluid velocity. It is because the thermal boundary layer gets thinner as the speed of fluid particles increases which promotes chaotic mixing because of which heat transfer increases. Figure 7a compares the Nusselt number of different types of ribs installed on all walls of MCHS, where MC-HR witnessed the best performance followed by MC-TR and MC-RR. The reason for achieving maximum Nusselt number by MC-HR is the streamlined surfaces both at leading and trailing edges of hexagonal ribs which offers a better contact of solid and fluid boundaries thus leading to better heat transfer performance. The reason for Nusselt number of MC-TR to be less than MC-HR is because of the absence of streamlining effect at the trailing edge of TR which causes flow to deviate from the rib surface. This flow deviation results in decreasing a better contact of solid fluid interface and also creating static fluid zones which causes reduction in local Nusselt number thus affecting average Nusselt number. When the edges of these ribs are chamfered by applying coning action on them, the Nusselt number gets decreased as shown in Fig. 7b. This reduction in Nusselt number is because of the reduction in effective heat transfer area as well the flow disturbance reduced by streamlining the edges of ribs. The thermal performance of MC-HR is significantly affected by coning action as compared to MC-TR and MC-RR because of its high number of edges whose surface area is greatly affected by coning action.

**Figure 8:**
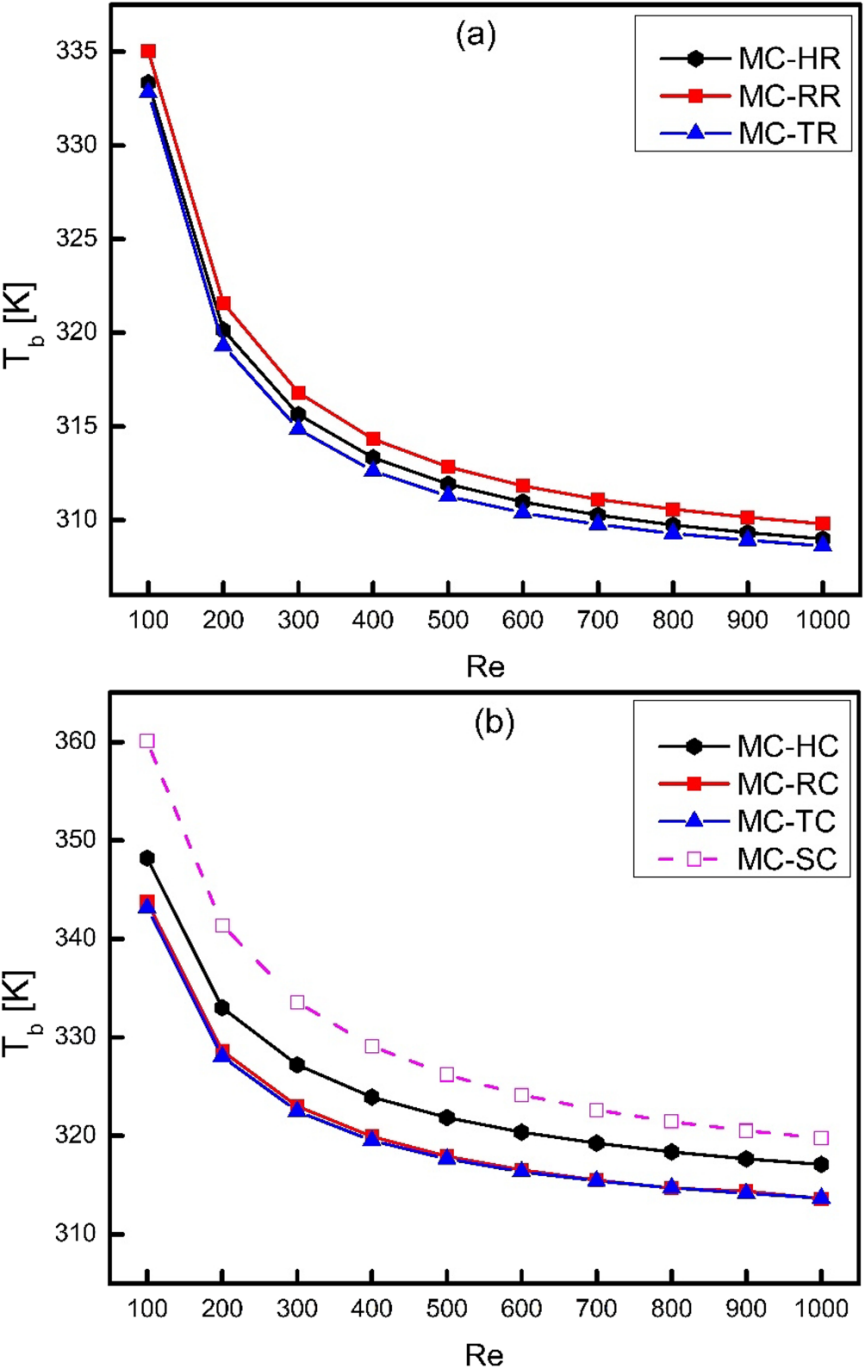
Variation of average base temperature of heat sink with Reynolds number.

The cooling performance of heat sink can be judged by comparing average base wall temperature for different types of ribs and cones. The higher value of T_b_ represents that lesser heat is conducted from heat sink to fluid whereas lower value of T_b_ represents a better conduction of heat to fluid. Figure 8 shows the comparison of average base wall temperature for different types of ribs and cones. It has been examined from Fig. 8a that MC-TR has witnessed minimum base wall temperature at all values of Reynolds number which means that triangular ribs have the ability to conduct heat easily as compared to rectangular and hexagonal ribs. When the ribs are converted into cones, their thermal performance has been compromised as shown in Fig. 8b. It is obvious that base wall temperature for all types of cones is higher than all types of ribs which means that thermal performance of ribs is better than cones. It is because the coning action has reduced the heat transfer area in case of cones which has reduced their Nusselt number and thus cooling has been minimized.

**Figure 9:**
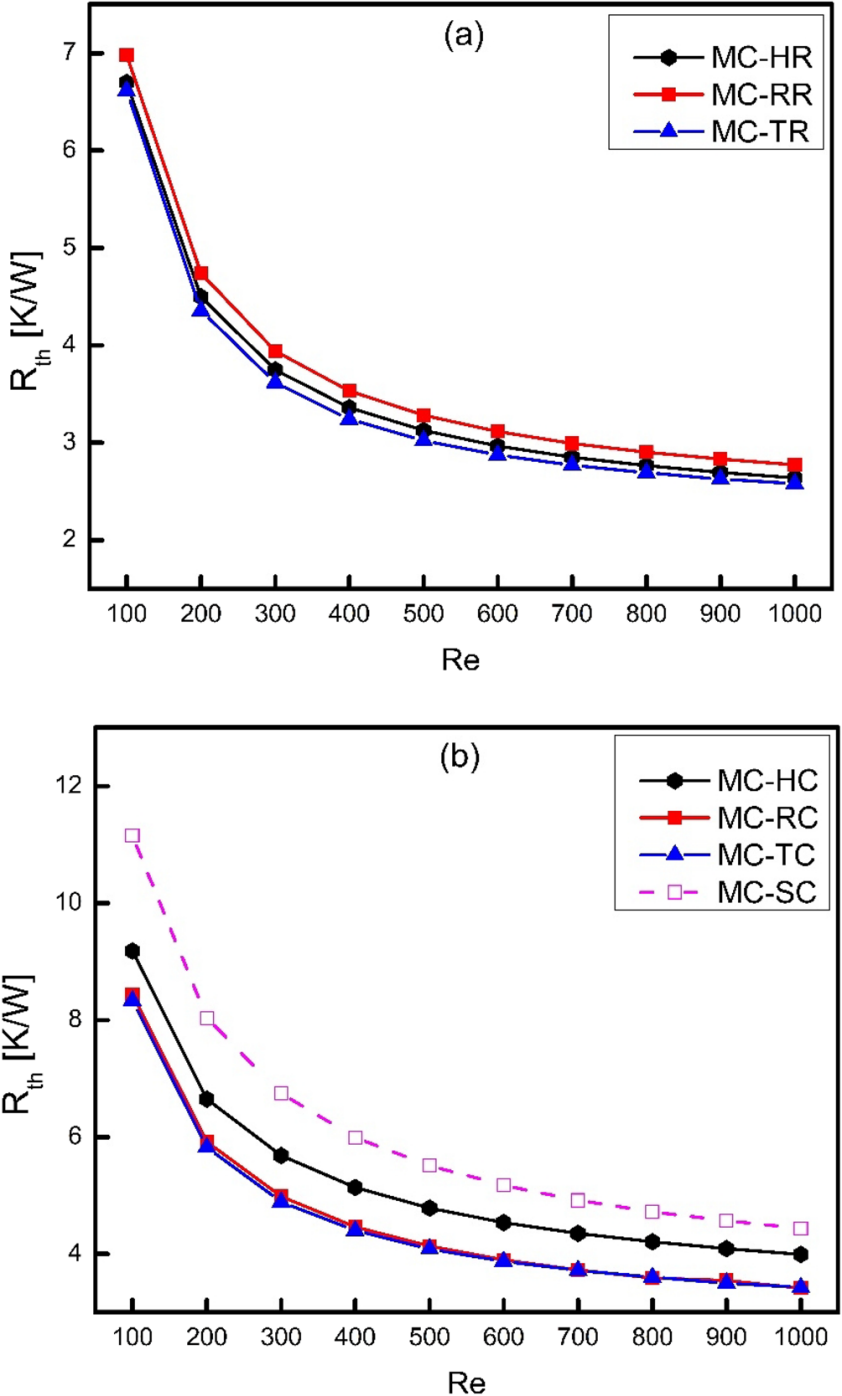
Variation thermal resistance with Reynolds number.

The analysis of thermal resistance plays a vital role in comparing the thermodynamics performance of systems. The higher is thermal resistance, the lower is the ability of system to transfer heat at a given temperature difference and vice versa. The comparison of thermal resistance for all types of ribs and cones has been shown in Fig. 9. It has been clearly shown that thermal resistance decreases as Reynolds number increases because the rate of heat transfer increases as the velocity of fluid increases at a given temperature difference. Moreover, thermal resistance of MC-RR is clearly higher than MC-TR and MC-HR as shown in Fig. 9a. It shows that rectangular ribs have the minimum capability of transferring thermal energy as compared to other ribs in the present study at a given temperature difference. Furthermore, it is also clear from the present study that the coning action has sacrificed for heat transfer performance due to which thermal resistance of cones has been increased as shown in Fig. 9b. The thermal resistance of MC-SC is maximum in all cases which shows the potential for improvement in its performance and it has been clearly done by adding ribs and cones.

### Entropy generation analysis

**Figure 10:**
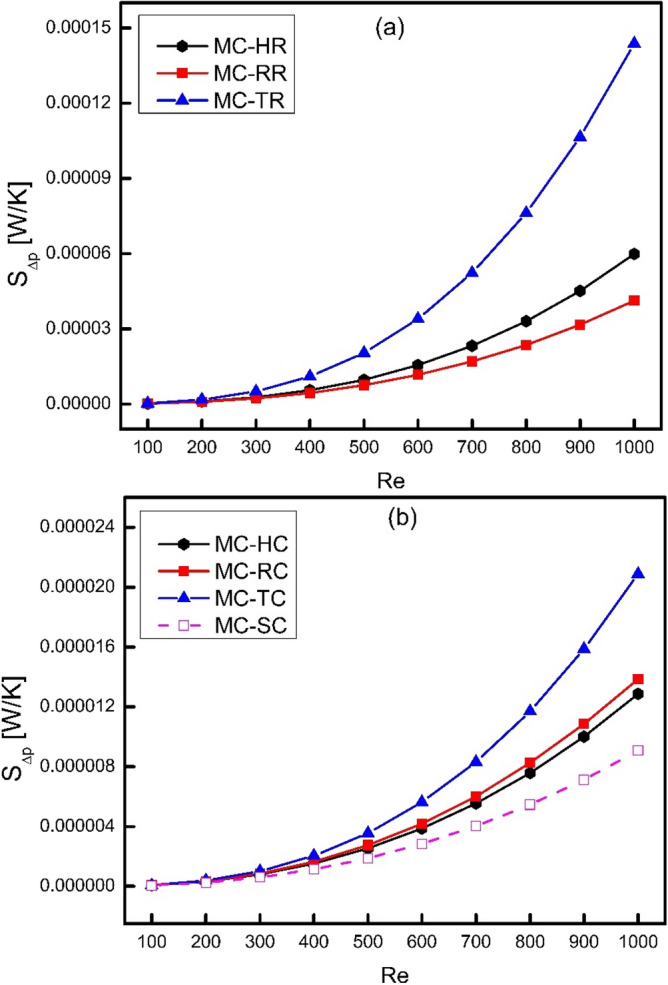
Variation of frictional entropy generation rate with Reynolds number.

When fluid flows inside a channel, there is always some losses because of friction in the form of pressure drop. Friction is an irreversible process where useful mechanical work is converted into an unused heat energy. Such type of frictional losses can be presented in the form of frictional entropy generation rate represented by ${\dot{S}}_{\Delta p}$ as shown in Fig. 10. It has been observed from the figure that frictional entropy generation rate increases with increase in Reynolds number because frictional losses are directly related to velocity of fluid. Moreover, it is also clear from Fig. 10a that triangular ribs have witnessed the maximum frictional entropy generation rate because of having more friction factor as evident from Fig. 5. Furthermore, it has been examined that the growth of trend is not linear but rather seems exponential and the trend of MC-TR is increasing faster than MC-RR and MC-HR. This fact can be attributed to the generation of vortices at the trailing edge of triangular ribs at higher Reynolds number which causes sudden pressure drop. Similarly, the frictional entropy generation rate of cones and smooth channel has been given in Fig. 10b. MC-TR has witnessed the highest frictional entropy generation rate followed by MC-RR and MC-HR and MC-SC.

**Figure 11:**
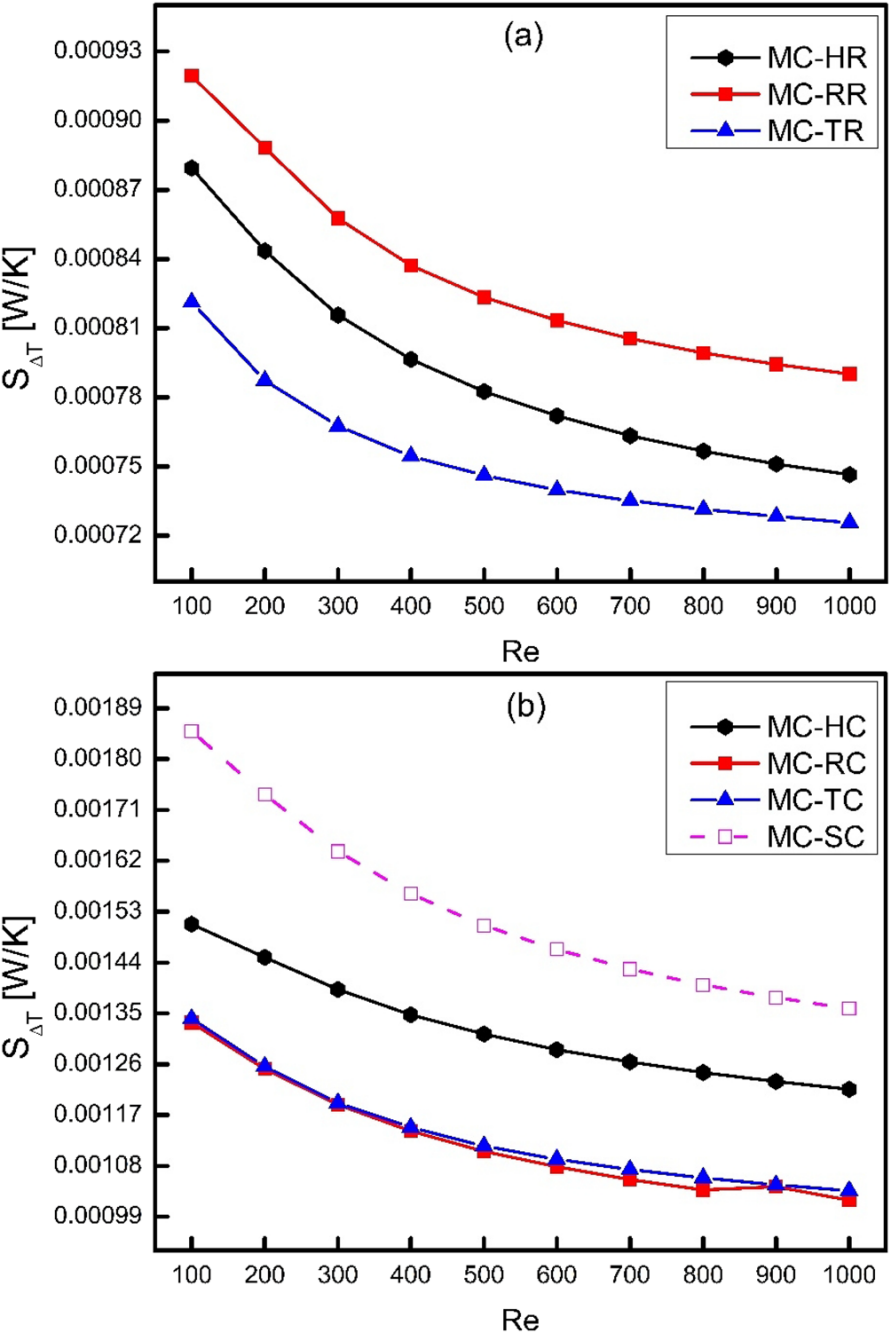
Variation of thermal entropy generation rate with Reynolds number.

For a thermal system where heat is required to be added to it or dissipated away from it, there is a need of driving force to flow this heat in a specific direction. This driving force is temperature difference and it is obvious that heat transfer always takes place in the direction of negative temperature gradient. Since the temperature difference is mandatory for heat transfer and the rate of heat transfer increases with increase in temperature difference. However, the higher temperature gradient causes the process to be more irreversible because of sudden change in temperature. Consequently, the higher temperature difference causes more entropy generation according to second law of thermodynamics. The irreversibility caused by heat transfer can be presented in the form of thermal entropy generation rate ${\dot{S}}_{\Delta T}$ as shown in Fig. 11. It is clear from the figure that thermal entropy generation rate decreases as the Reynolds number increases because the temperature difference between channel wall and fluid decreases with increase in fluid velocity. However, the rate of heat transfer increases because of the rapid increase in heat transfer coefficient. Figure 11a compares thermal entropy generation rate for different types of ribs which shows that triangular ribs has the minimum thermal losses as compared to rectangular and hexagonal ribs. Moreover, Fig. 11b compares thermal entropy generation rate for different types of cones. It has been examined that thermal losses of cones are higher than that of ribs because of the reduction in heat transfer coefficient and increase in temperature difference between solid and fluid domains. However, similar to triangular ribs, thermal entropy generation rate of triangular cones is minimum as compared to other cones and smooth channel. It is also clear from Fig. 11 that MC-SC has maximum thermal losses and therefore shows the need of ribs and cones to cause reduction in thermal losses.

**Figure 12:**
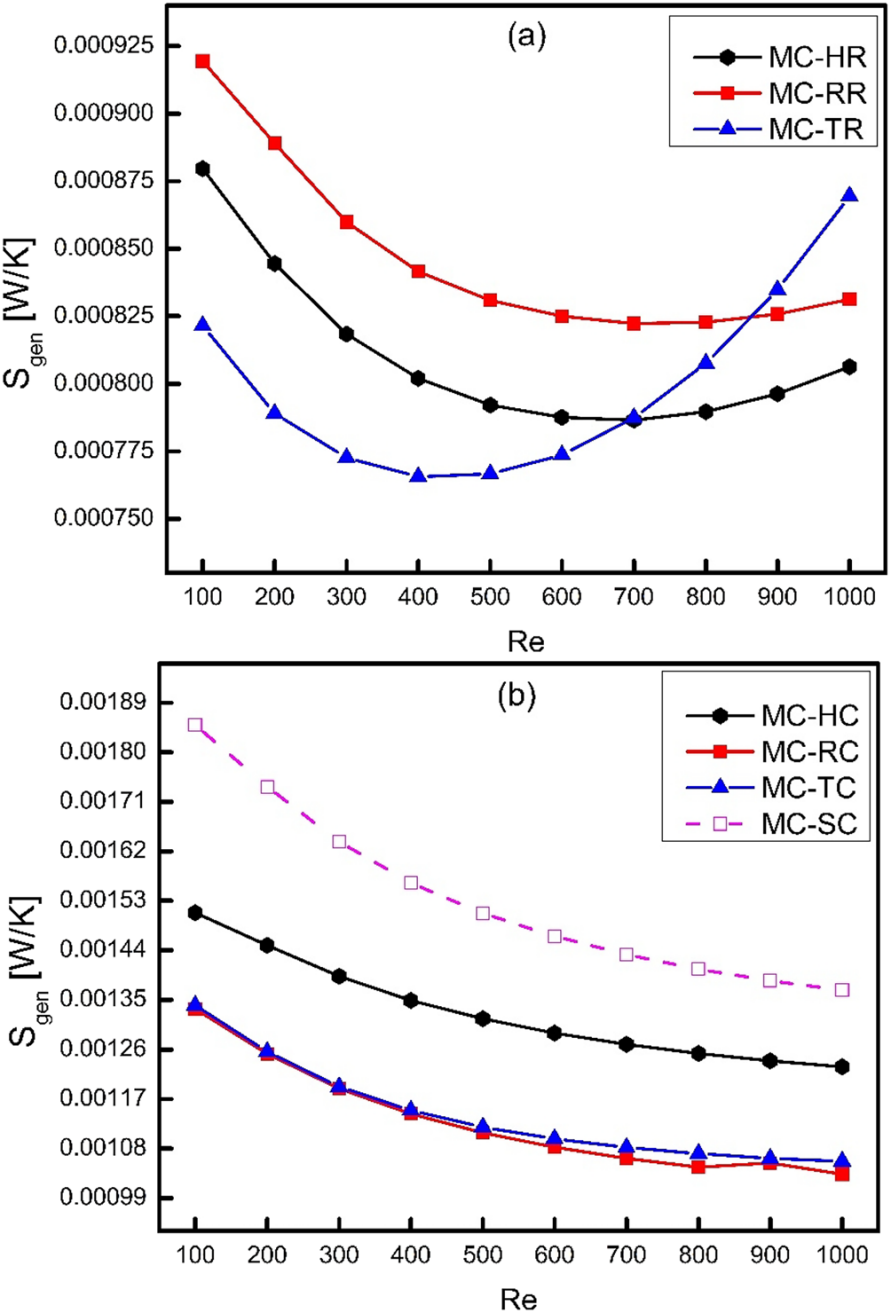
Variation of total entropy generation rate with Reynolds number.

As the Reynolds number increases, the frictional entropy generation rate increases whereas thermal entropy generation rate decreases. Moreover, the frictional losses get decreased as a result of coning whereas thermal losses get increased because coning decreases both pressure drop and heat transfer coefficient. Therefore, the total entropy generation rate is required to be calculated for the comparison of different cases considered in the present study. Figure 12 shows the comparison of total entropy generation rate for different types of ribs and cones. It has been clearly observed from Fig. 12a and b that all types of ribs have entropy generation less than all types of cones and smooth channel. Moreover, from Fig. 12a, another interesting result can be achieved that the trend of MC-TR decreases up to Re = 400 and then increases onwards. It means that at lower Reynolds number, thermal losses are more significant than frictional losses. However, at higher Reynolds number, where vortex generation takes place especially in MC-TR, frictional losses dominate over thermal losses. Furthermore, it has been examined that frictional losses are very lower that thermal losses in case of cones, which means that thermal losses are more significant in such systems. As a final remark, it can be recommended for future designs to more focus on thermal losses in such systems where no vortex generation or local turbulence can take place. However, in order to minimum the total entropy generation in thermodynamic systems where chaotic mixing, turbulence and vortex generation takes place at higher Reynolds number, there should be a critical analysis for both frictional and thermal losses.

**Figure 13:**
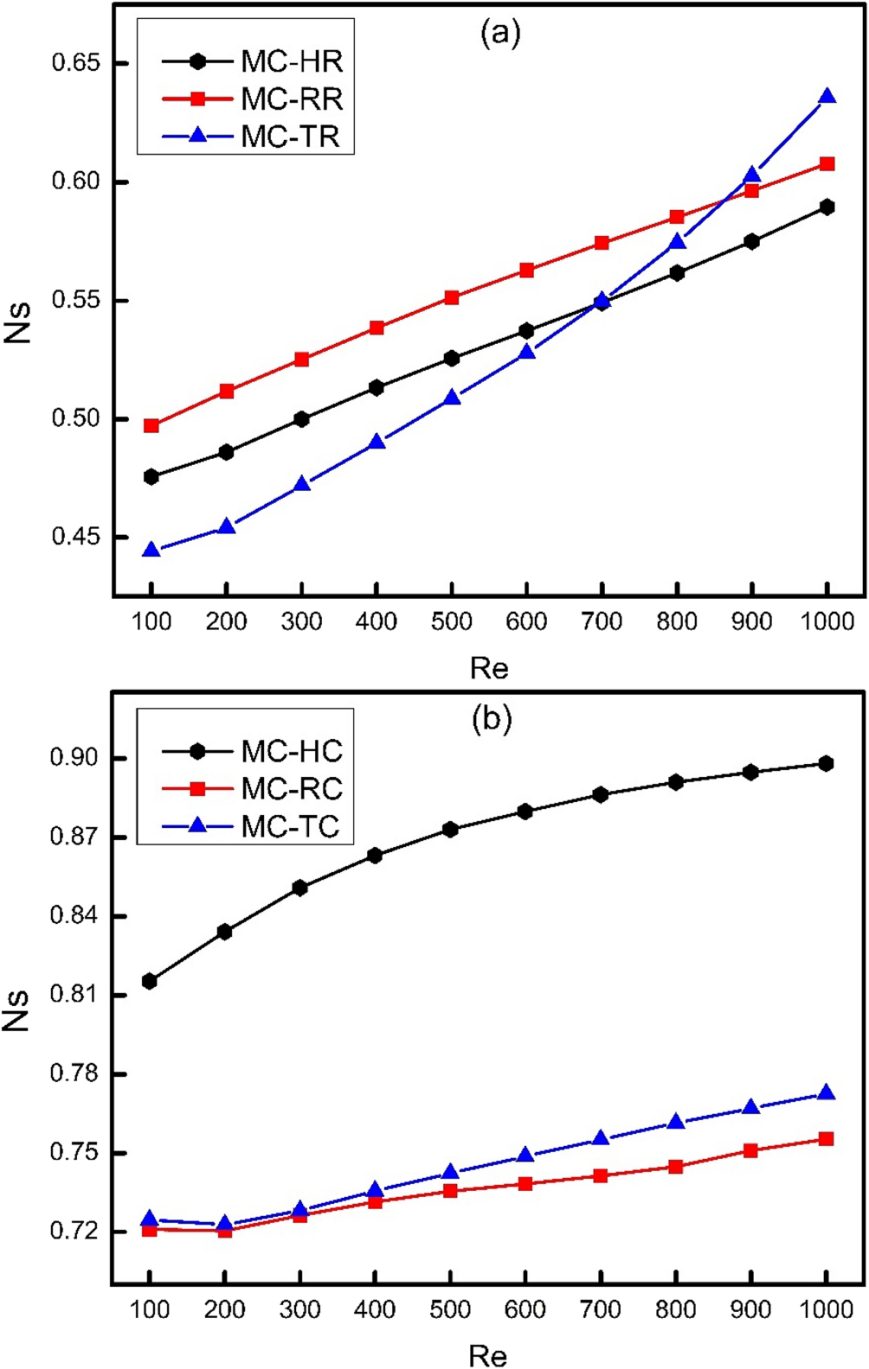
Variation of augmentation entropy generation number with Reynolds number.

To compare the total entropy generation rate of enhanced channel with that of smooth channel, the concept of augmentation entropy generation number (Ns) can be used. Augmentation entropy generation number is the ratio of entropy generation of any channel to that of smooth channel. For any enhanced channel to be thermodynamically better than its referenced smooth channel, its augmentation entropy generation number must be less than unity. The lesser is the value of Ns for any channel, the better is that channel than smooth channel. Figure 13 shows the comparison of augmentation entropy generation number for different types of ribs and cones. It is clearly shown in Fig. 13 that augmentation entropy generation number increases with increase in Reynolds number because the entropy generation of MC-SC is decreasing more rapidly than all the other channels. It means that the losses are of enhanced channels get more prominent at higher Reynolds number. Moreover, the augmentation entropy generation number of MC-TR is minimum in all cases at Re < 700 and after Re > 700, MC-HR has the lowest value of Ns. It is because the total entropy generation rate for MC-TR decreases up to Re = 400 and then increases after Re = 400 and finally at Re = 700, it becomes higher than MC-HR as shown in Fig. 12a. It is also obvious from Fig. 13a and b that augmentation entropy generation number of ribs is lower than cones which is also clear from Fig. 12. Furthermore, it can be seen from Fig. 13b that MC-HC has the highest augmentation entropy generation number.

### Exergy analysis

**Figure 14:**
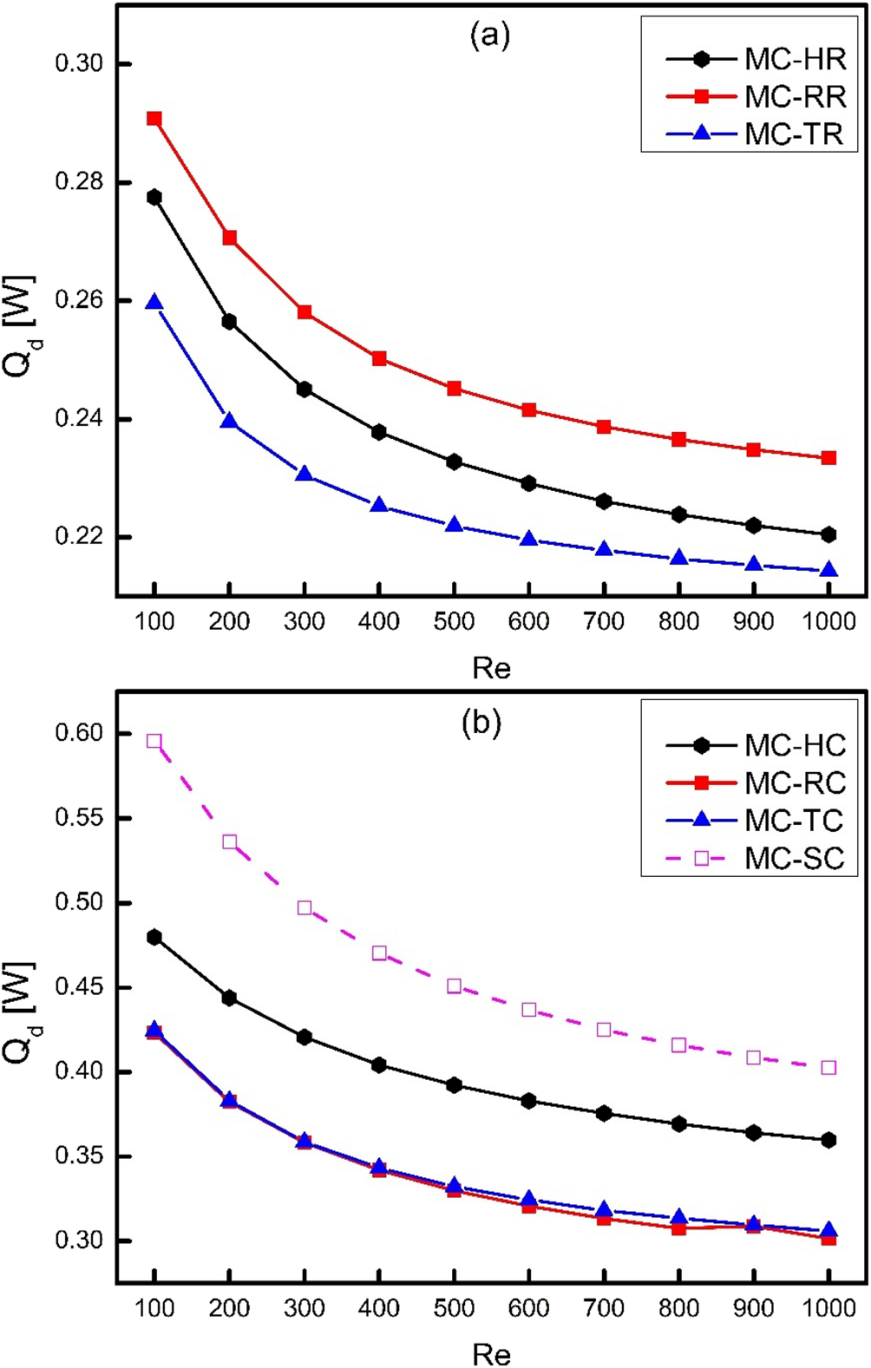
Variation of irreversible heat loss with Reynolds number.

Since the thermal resistance of channel is due to the net temperature gradient, therefore, there is some amount of heat loss which is called as irreversible heat loss (Q_d_). Figure 14 shows the variation of irreversible heat loss for different types of ribs and cones as a function of Reynolds number. It is obvious that irreversible heat loss decreases with increase in Reynolds number because of the decrease in net temperature gradient. It is also clear that MC-TR has the minimum irreversible heat loss because it has the lowest thermal resistance and base wall temperature as shown in Figs. 8 and 9. Moreover, the maximum irreversible heat loss is occurred in MC-SC because of the worst heat transfer behavior which also showed the need to enhance the channel. Furthermore, it can be seen from Fig. 14 that the coning action lead to increase the irreversible heat loss because of increased thermal resistance and entropy generation.

**Figure 15:**
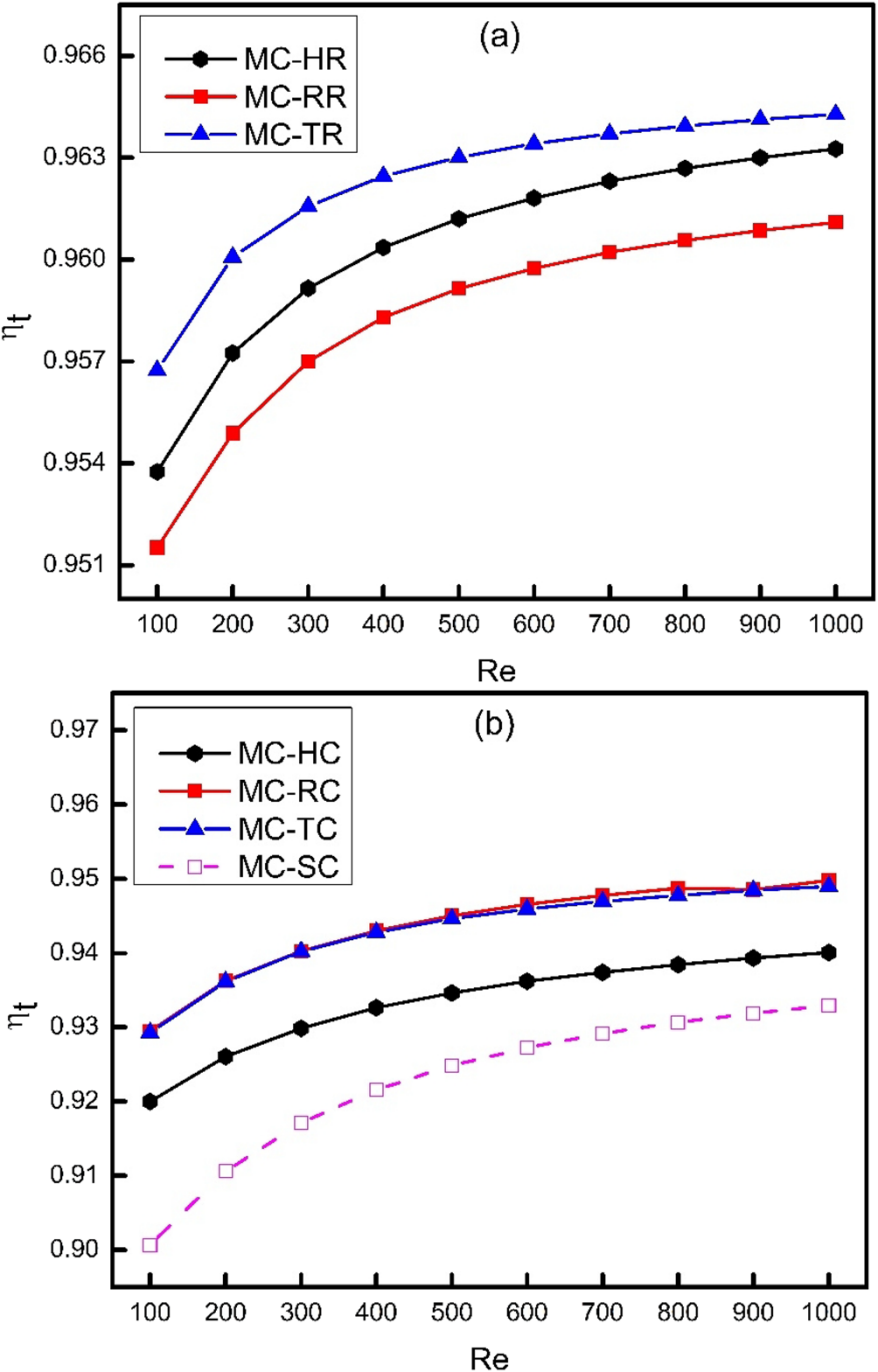
Variation of transport efficiency with Reynolds number.

To utilize the result of irreversible heat loss, transport efficiency of thermal energy (ƞ_t_) can be defined which can be considered as the exergy (available energy) of thermal system. It is obvious that transport efficiency has inverse relation with irreversible heat loss as can be seen from Figs. 14 and 15. The effect of increasing Reynolds number on the variation of transport efficiency of thermal energy has been shown in Fig. 15. It is clearly shown that transport efficiency of thermal energy increases with increase in Reynolds number which is because of the reduction in net temperature gradient at higher fluid velocities. Due to this decrease in net temperature gradient, the irreversibility get decreases which results in better utilization of thermal energy in transporting heat away from the system which is the ultimate goal of this study. However, an interesting result has been observed from Fig. 15 that the slope of graph decreases with increase in Reynolds number and almost becomes zero at Re = 1000 which shows that further increase in Reynolds number will not help in improving thermal performance of MCHS. It can also be seen from Fig. 15a and b that ribs showed a better transport of thermal energy than cones. Moreover, it has been examined that MC-TR has witnessed the maximum transport efficiency which is 96.3% at higher Reynolds number.

## Conclusions

In the present study, three dimensional numerical simulations have been done to investigate the effect of ribs on thermodynamic behavior of MCHS in a laminar flow of Reynolds number varying from 100 to 1000. Moreover, the ribs were drafted at an angle of 450 to reduce their drage to improve the hydrodynamic performance of MCHS. The results of this study are expected to provide a guideline for future recommendations of MCHS design on the basis of first and second law of thermodynamics. Some of the major conclusions that have been drawn from the present study can be presented as follows:

1. The application of novel effect of coning at an angle of 45^0^ lead to reduce frictional losses by streamlining the ribs to reduce blocking effect, however; a compromise on thermal behavior has been shown. Moreover, the application of coning has caused a significant reduction in wall shear stress and friction factor which can lead to reduce the pumping power requirements of MCHS. Similarly, due to reduction in friction factor and wall shear stress caused by coning of ribs, there is a significant amount of reduction in frictional entropy generation rate.
2. The comparison of Nusselt number for different types of ribs and cones showed that MC-HR has witnessed the best performance because of the streamlined surfaces both at leading and trailing edges of hexagonal ribs which offers a better contact of solid and fluid boundaries thus leading to a better heat transfer performance. The Nusselt number of MC-TR was examined to be less than MC-HR because of the absence of streamlining effect at the trailing edge of TR which causes flow to deviate and thus decreasing a better contact of solid fluid interface. When the edges of these ribs are chamfered by applying coning action on them, the Nusselt number gets decreased because of the reduction in effective heat transfer area as well the flow disturbance reduced by streamlining the edges of ribs.
3. It has been examined that MC-TR has witnessed minimum base wall temperature at all values of Reynolds number in the present study which means that triangular ribs have the ability to conduct heat easily as compared to rectangular and hexagonal ribs.
4. It has been observed that frictional entropy generation rate increases with increase in Reynolds number because frictional losses are directly related to velocity of fluid. Moreover, it has been examined that the growth of trend is not linear but rather seems exponential and the trend of MC-TR is increasing more rapidly than MC-RR and MC-HR. This fact can be attributed to the generation of vortices at the trailing edge of triangular ribs at higher Reynolds number which causes sudden pressure drop.
5. With the increase in Reynolds number, the frictional entropy generation rate increases whereas thermal entropy generation rate decreases. Moreover, the frictional losses get decreased as a result of coning whereas thermal losses get increased. Therefore, the total entropy generation rate is to be calculated to combine the effect of both. It has been examined in the present study that the trend of total entropy generation rate for MC-TR decreases up to Re = 400 and then increases onwards which means that thermal losses are more significant than frictional losses at lower Reynolds number. However, frictional losses dominate over thermal losses at higher Reynolds number, where vortex generation takes place, especially in MC-TR. Furthermore, it has been examined that frictional losses are much lower than thermal losses in case of cones, which means that thermal losses are more significant in such systems.
6. As a final remark, it can be recommended for future designs to more focus on thermal losses in such systems where no vortex generation or local turbulence takes place. However, there should be a critical analysis for both frictional and thermal losses to evaluate the total entropy generation in thermodynamic systems where chaotic mixing, turbulence, and vortex generation take place.

## Data Availability

All the data is available in this paper. There is no supplemental data used in this paper.

## List of symbols

${L}_{t}$: Total length of the channel, m
$$A$$: Area, m^2^
$$H$$: Height, m
${D}_{h}$: Hydraulic diameter of the channel, m
$$W$$: Width, m
${c}_{p}$: Specific heat, J kg^-1^ K^-1^
$$q$$: Heat flux, W.m^-2^
$$k$$: Thermal conductivity, W m^-1^ K^-1^
$\dot{m}$: Mass flow rate, kg/sec
$h$: Heat transfer coefficient, W m^-2^ K^-1^
$u,v,w$: Velocity components in x, y, and z directions, m s^−^^1^
$${u}_{m}$$: Mean velocity
$$\nabla T$$: Temperature gradient, K
$\nabla p$: Pressure gradient, Pa
$$Re$$: Reynolds number
$${P}_{O}$$: Poiseuille number
$$Pr$$: Prandtl number
$$E$$: Error
${Nu}_{0}$: Nusselt number of smooth channel
$Nu$: Nusselt number of enhanced channel
${f}_{0}$: Friction factor of smooth channel
$f$: Friction factor of enhanced channel
${\dot{Q}}_{d}$: Irreversible heat loss, W
${\dot{S}}_{gen, \Delta T}$: Entropy generation due to heat transfer, W/K
${\dot{S}}_{gen, \Delta p}$: Entropy generation due to friction, W/K
$\dot{S}^{\prime\prime\prime}_{gen}$: Volumetric Entropy generation, W/m^3^ K
${\dot{S}}_{gen}$: Total Entropy generation, W/K
${N}_{s}$: Augmentation entropy generation number
$\Delta p$: Pressure difference, Pa
$$\Delta T$$: Temperature difference, K
R_th_: Thermal resistance, K/W

## Abbreviations

MCHS: Microchannel heat sink
MC-TR: Microchannel with triangular ribs
MC-HR: Microchannel with hexagonal ribs
MC-RR: Microchannel with rectangular ribs
MC-TC: Microchannel with triangular cones
MC-HC: Microchannel with hexagonal cones
MC-RC: Microchannel with rectangular cones
AR: Aspect ratio of the channel

## Greek letters

$$\rho$$: Density, kg m^-3^
$\mu$: Dynamic viscosity, Pa s
$${\upeta }_{t}$$: Transport efficiency
$\upeta$: Thermal enhancement factor

## Subscripts

$s$: Solid (Except in N_s_)
$$f$$: Fluid
$$w$$: Wall
$$ch$$: Channel
$$in$$: Inlet
$$out$$: Outlet
$$t$$: Total
